# On the Symmetry, Electronic Properties, and Possible Metallic States in NASICON-Structured A_4_V_2_(PO_4_)_3_ (A = Li, Na, K) Phosphates

**DOI:** 10.3390/ma16124361

**Published:** 2023-06-13

**Authors:** Denis Gryaznov, Linas Vilčiauskas

**Affiliations:** 1 Center for Physical Sciences and Technology (FTMC), Saulėtekio al. 3, LT-10257 Vilnius, Lithuania; 2 Institute of Solid State Physics, University of Latvia, Kengaraga 8, LV-1063 Riga, Latvia

**Keywords:** density functional theory, hybrid-exchange-correlation functionals, NASICON, sodium vanadium phopshate

## Abstract

In this work, the electronic structure and properties of NASICON-structured A_4_V_2_(PO_4_)_3_, where A = Li, Na, K were studied using hybrid density functional theory calculations. The symmetries were analyzed using a group theoretical approach, and the band structures were examined by the atom and orbital projected density of states analyses. Li_4_V_2_(PO_4_)_3_ and Na_4_V_2_(PO_4_)_3_ adopted monoclinic structures with the C2 space group and averaged vanadium oxidation states of V${}^{+2.5}$ in the ground state, whereas K_4_V_2_(PO_4_)_3_ adopted a monoclinic structure with the C2 space group and mixed vanadium oxidation states V${}^{+2}$/V${}^{+3}$ in the ground state. The mixed oxidation state is the least stable state in Na_4_V_2_(PO_4_)_3_ and Li_4_V_2_(PO_4_)_3_. Symmetry increases in Li_4_V_2_(PO_4_)_3_ and Na_4_V_2_(PO_4_)_3_ led to the appearance of a metallic state that was independent of the vanadium oxidation states (except for the averaged oxidation state R32 Na_4_V_2_(PO_4_)_3_). On the other hand, K_4_V_2_(PO_4_)_3_ retained a small band gap in all studied configurations. These results might provide valuable guidance for crystallography and electronic structure investigations for this important class of materials.

## 1. Introduction

The ongoing search for new ion insertion materials with superior characteristics is one of the central themes in materials and electrochemical science [1]. They are at the heart of active electrode materials in batteries, desalination cells, and many other electrochemical devices. Na SuperIonic Conductor (NASICON) structure type phosphate frameworks with a general formula of A_x_M_2_(PO_4_)_3_ (where *x* is from 1 to 4, A is typically an alkali metal such as Li, Na, and K, and  M is a transition metal, e.g., Ti, V, Cr, Fe, Mn, Co, Ni etc., or their combinations) are attracting a lot of attention [2,3,4]. This is due to their structural diversity, wide range of available redox potentials, electrochemical stability and reversibility, and high ionic mobility. These properties are useful not only for alternative battery technologies, but also for other electrochemical applications such as Faradaic deionization/desalination cells [5,6,7,8,9,10].

Further progress in this field still requires a lot of fundamental understanding as well as materials engineering in order to fully realize the potential of such framework materials. Despite a substantial theoretical effort, there is still a very limited understanding of the electronic structure and chemical bonding in these systems at an atomic level. In fact, it is these effects that really govern the material properties and behavior during electrochemical operation. These include the formation and mobility of ionic and polaronic charge carriers, interactions and ordering within the alkali, or transition metal sublattices, variation in the electronic band structure during ion insertion process etc. [11,12,13,14,15].

The analysis presented in this work was inspired by some of our recent results and the work of Wang et al. [16]. These results are based on Density Functional Theory (DFT) calculations of a fully sodiated Na_4_V_2_(PO_4_)_3_ composition and indicate the intriguing possibility of an appearance of a metallic state in this particular phase. It is important to mention that NASICON-structured materials similar to other polyanionic framework compounds are typically assumed to be wide band gap semiconductors with a very low electronic charge carrier concentration and conductivity. In practice, there is a lot of additional processing and additives necessary when using these materials as electrodes. The potential existence of an unexpected metallicity in this class of materials could not just open new possibilities, but also would be very important to understand from a fundamental point of view.

The NASICON structure consists of an open framework of corner-sharing VO_6_ octahedra and PO_4_ tetrahedra with two different sites available for alkali metal occupation. At ambient conditions, NASICON compounds are typically assumed to adopt a high symmetry rhombohedral structure with a $R\bar{3}c$ (No. 167) space group (SG) (γ-phase). This is naturally expected in Na_1_M_2_(PO_4_)_3_ and Na_4_M_2_(PO_4_)_3_ structures, which both have fully occupied M1 or Na1 sites and either empty or fully occupied M2 or Na2 sites in the NASICON structure, respectively. Although recently, the second-order Jahn–Teller effect has been shown to exist and lead to the transition to a $R\bar{3}$ space group in NASICON systems with *x* = 1 [17]. However, for the intermediate occupation of M2 positions in Na_2_M_2_(PO_4_)_3_ and Na_3_M_2_(PO_4_)_3_ compositions, the situation is more complex. For example, Na_3_V_2_(PO_4_)_3_, due to Na disorder, adopts a R$\bar{3}$c space group only above 177.2 °C [12], but, at lower temperatures sodium ordering and symmetry reduction is frequently observed [11,12,15,16].

The primitive unit cell of a fully occupied Na_4_M_2_(PO_4_)_3_ consists of 42 atoms (*Z* = 2): 8 Na, 4 M, 6 P, and 24 O atoms. Therefore, Na atoms are distributed among 2 M1 or Na1 (Wyckoff position: 6b (0,0,0)) and 6 M2 or Na2 (Wyckoff position: 18e$\left(x,0,\frac{1}{4}\right)$) positions in the primitive unit cell. The position multiplicity in the conventional unit cell contains a three times larger number of atoms, i.e., *Z* = 6. In addition, oxygen ions occupy two low symmetry Wyckoff positions 36f with different coordinates, whereas vanadium ions occupy the Wyckoff position 12c.

The local symmetry of a transition metal site also introduces another degree of freedom in terms of possible symmetries and sublattice ordering (also known as the Verwey-type ordering) in a NASICON structure. Even with only one type of metal is occupying the site, it might adopt a mixed-valence (or mixed-oxidation state) state due to charge neutrality requirements or disproportionation [13,18]. Throughout this work, the following nomenclature will be used for referring to the oxidation states (OSs) of vanadium in A_4_V_2_(PO_4_)_3_. The state of all vanadium atoms adopting equal fractional valence oxidation state of V${}^{+2.5}$ will be interchangeably referred as averaged oxidation (valence), or non-mixed-valence state. The case where half of the vanadium atoms have V${}^{+2}$ and the other half V${}^{+3}$ oxidation states will be referred to as mixed-oxidation or mixed-valence state, interchangeably.

The symmetry and orderings in the alkali and transition metal sublattices are strongly dependent on the particular metal composition. Moreover, the experimental investigation of these effects is highly non-trivial, because high-intensity X-ray sources and a careful control of the sample preparation and measurement conditions are necessary [11].

In this study, we presented a comprehensive electronic structure analysis of NASICON-structured A_4_V_2_(PO_4_)_3_ systems, where A = Li, Na, K. We used high-level hybrid density functional theory calculations, the group theoretical symmetry approach, and the projected density of states analyses in this work. The structures with different alkali metals in the NASICON structure were fully optimized and analyzed in terms of available symmetries and band structure. A comparative analysis in terms of different-sized alkali metal cations by correspondingly replacing sodium with lithium and potassium was also performed. To the best of our knowledge, this is one of the first of such studies where the electronic aspects have been addressed in this class of materials at this level of detail and complexity.

## 2. Computational Methodology

Density Functional Theory (DFT) within Linear Combination of Atomic Orbitals (LCAO) formalism, together with B1WC hybrid-exchange-correlation functionals as implemented in the CRYSTAL17 computer program, were used in this work [19,20]. All-Electron Gaussian basis sets of Triple-Zeta Valence with Polarization (TZVP) functions on Na, P and O were taken from Peintinger et al. [21]. In contrast, revised versions of pob-TZVP basis sets for Li, K, and V were taken from Vilela-Oliveira et al. [22]. The number of contracted basis functions (s/p/d/f) was 73211/511/1 for Na, 62111 for Li, 842111/6311/1 for K, 842111/6311/411/1 for V, 73211/5111/1 for P, and 6211/411/1 for O. The Monkhorst–Pack scheme was applied to select the *k* points on a 8 × 8 × 8 mesh for the integration of the first Brillouin zone in spin-polarized calculations of primitive unit cells [23]. The Coulomb and exchange integral tolerance factors of 8, 8, 8, 8, and 16 were used in the calculations, together with an extra large numerical integration grid. The energy convergence threshold was set to 10${}^{-8}$ a.u. for the Self-Consistent Field (SCF) of total energy and 10${}^{-7}$ a.u. for the optimization of lattice parameters and atomic positions. We stress the importance of selecting an appropriate hybrid-exchange-correlation functional for the approximate treatment of electronic correlation effects in transition metal compounds [24]. It is well-known that standard Generalized-Gradient Approximation (GGA) exchange-correlation functionals underestimate the band gap and are unable to reproduce the correct electronic configuration, together with the associated structural distortions in transition metal compounds [24,25]. Hybrid-exchange-correlation functionals effectively combine Hartree–Fock (HF) and DFT approaches, which yield significantly better results and more accurate properties [24]. Wu and Cohen proposed a non-empirical exchange-correlation functional with an improved exchange part of the standard PBE density functional (denoted as WC functional) [26]. Bilc et al. [25] combined WC functional with hybrid B1 functional and a 0.16 mixing parameter for the exact exchange into B1WC hybrid-exchange-correlation functional. All the calculations were spin-polarized while assuming high spin states for V${}^{+2}$ [3d${}^{3}$] and V${}^{+3}$ [3d${}^{2}$]. The effective atomic charges and magnetic moments were calculated from the Mulliken population analysis.

## 3. Results and Discussion

### 3.1. Group Theoretical Approach to NASICON Structures

**Figure 1:**
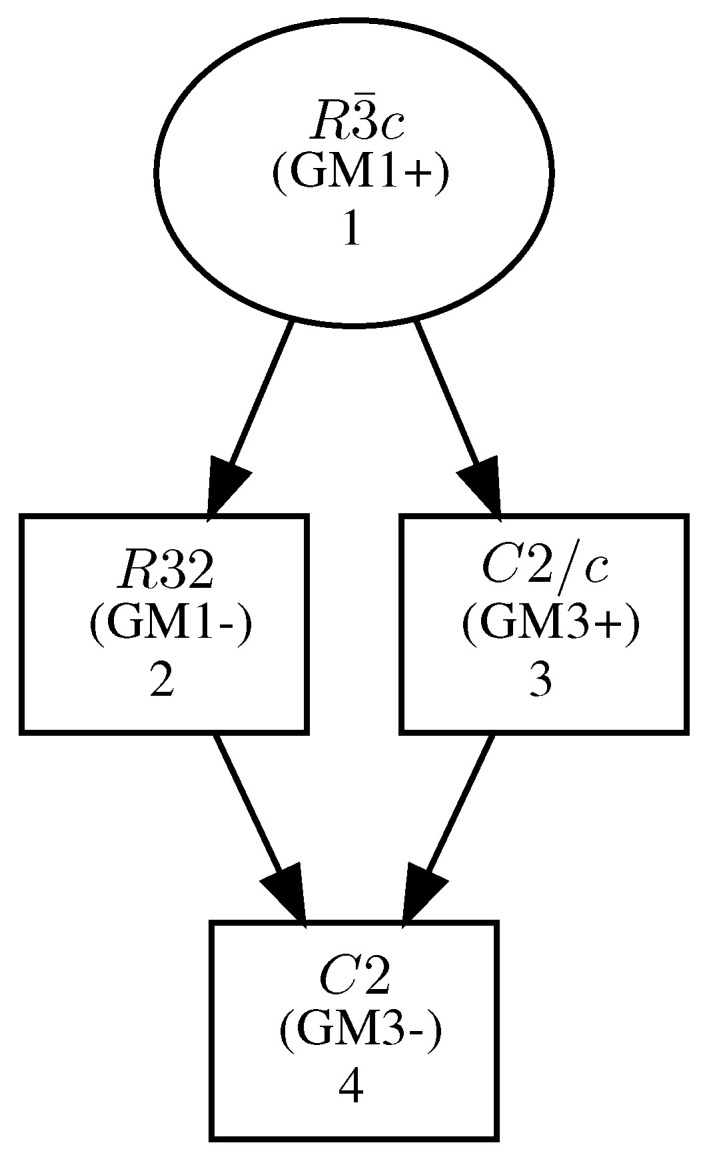
Graph of isotropy subgroups relating $R\bar{3}c$ and C2 [29].

In the present study, we relied on crystallographic group theory and group-subgroup relations in order to construct low symmetry structures of A_4_V_2_(PO_4_)_3_ as well as to describe changes in the electronic structure appearing upon symmetry reduction. It is worth mentioning that such an approach is necessary, but not sufficient, to infer possible phase transitions. We also did not attempt to find the lowest possible symmetry, but mostly focused on the relationship between symmetry and total energy. The maximal subgroup approach provided a list of maximal subgroups for $R\bar{3}c$, which included C2/c, R32, $R\bar{3}$, R3c, and $P\bar{3}c1$ [27]. For simplicity, we limited ourselves to the monoclinic C2 SG and chose one chain of transitions. In this way, we suggest that considering only C2 SG is sufficient for discussing potential phase transitions to low symmetry phases in the present system. Among the maximal subgroups of $R\bar{3}c$, there were only two SGs: R32 and C2/c, which were both leading to C2. Moreover, the group-maximal subgroup tree (similar to the Bärnighausen tree) contained only two simple chains from $R\bar{3}c$ to C2, i.e., R$\bar{3}$c →C2/c→C2 and $R\bar{3}c\to R32\to C2$ [28]. Moreover, the analysis of isotropy subgroups revealed that the $R\bar{3}c$ → R32 transition was determined by irreducible representation $\Gamma_{1}^{-}$, whereas the R32 → C2 transition took place according to the primary irreducible representation $\Gamma_{3}^{-}$ (Figure 1). In this work, the chain through R32 was chosen and used in the following procedure. At first, the system was relaxed in a high symmetry $R\bar{3}c$ supergroup; then, necessary symmetry operations were removed in order to relax the system in a lower symmetry rhombohedral structure (subgroup R32); finally, the necessary symmetry operations were removed from a relaxed lower symmetry rhombohedral structure (supergroup R32) in order to relax to the system in a low symmetry monoclinic structure (subgroup C2).

### 3.2. Analysis of the Low Energy Structures

Having constructed structures with different symmetries, we proceeded to calculate and rank their energies in order to identify the ones with the lowest energies. At this point, it is worth noting that mixed OS cannot be obtained in a high symmetry $R\bar{3}c$ SG. R32 is the highest possible symmetry where mixed OS can be obtained in A_4_V_2_(PO_4_)_3_. In this case, two vanadium Wyckoff positions, which were of the same symmetry in $R\bar{3}c$ SG, split under symmetry reduction into two different Wyckoff 6c positions in the R32 SG.

The total energies of the structures with different SGs and OSs are compared with respect to the lowest energy structure (ground state) in Table 1. The ground state configuration for Li_4_V_2_(PO_4_)_3_ and Na_4_V_2_(PO_4_)_3_ adopted monoclinic symmetry with a C2 SG and an averaged vanadium OS (V${}^{+2.5}$). This was different from the case of K_4_Ti_2_(PO_4_)_3_, which adopted mixed OSs (V${}^{+2}$/V${}^{+3}$), with C2 as the lowest energy structure. This indicates the importance of alkali metal on the structural and, as it is shown later, on the electronic properties of such compounds [13]. The relative energy differences between the lowest and higher energy structures were comparable for Li_4_V_2_(PO_4_)_3_ and Na_4_V_2_(PO_4_)_3_. The structures in the $R\bar{3}c$ SG and the averaged V${}^{+2.5}$ OS were 0.597 eV/cell and 0.595 eV/cell higher in energy for Li_4_V_2_(PO_4_)_3_ and Na_4_V_2_(PO_4_)_3_, respectively. The R32 structures and mixed V${}^{+2}$/V${}^{+3}$ OSs were 0.94 eV/cell and 0.825 eV/cell less favorable than the averaged OS C2 ones for Li_4_V_2_(PO_4_)_3_ and Na_4_V_2_(PO_4_)_3_, respectively. Also, the R32 structure with the averaged OS was the second lowest energy structure in both Na_4_V_2_(PO_4_)_3_ and Li_4_V_2_(PO_4_)_3_. The important finding here is that, identically to the previous results of Wang et al. [16] using the SCAN+U approach, the B1WC hybrid functional also showed that Li_4_V_2_(PO_4_)_3_ and Na_4_V_2_(PO_4_)_3_ structures with averaged OSs were lower in energy than those with a mixed valence. The hypothetical phase transition from $R\bar{3}c$ to R32 would correspond to the charge ordering transition within the mixed oxidation V${}^{+2}$/V${}^{+3}$ sublattice without any accompanying A^+^-vacancy ordering due to full alkali metal sublattice occupancy.

In contrast to Na- and Li- based A_4_V_2_(PO_4_)_3_ NASICON structures, the C2 SG and mixed V${}^{+2}$/V${}^{+3}$ OS had the lowest energy in K_4_V_2_(PO_4_)_3_ (Table 1). This suggests that symmetry reduction down to monoclinic symmetry was favorable in all three studied systems. Moreover, the energy differences between different symmetry configurations were a bit smaller in K_4_V_2_(PO_4_)_3_ than in the other two cases. K_4_V_2_(PO_4_)_3_ with the C2 SG and averaged V${}^{+2.5}$ OS was only 0.200 eV/cell, and with $R\bar{3}c$, its averaged OS was only 0.153 eV/cell less stable than the ground state structure.

The results suggest that it is not only the overall symmetry reduction and the type of vanadium OS that have an effect on the structure and stability of these compounds. The ionic size, and potentially the electronic structure of the ion at the alkali metal site, also seem to play an important role.

### 3.3. Electronic Structure Analysis

The electronic structure and properties of the A_4_V_2_(PO_4_)_3_ system are the main focus of the present study. Table 1 summarizes the calculated Mulliken effective atomic charges (*q*) and magnetic moments (μ) for vanadium ions in all studied structures. As expected, the charge values were correlated with the formal OS. There was also a clear correlation between the V charge in structures with different alkali metals: Li compounds always showed lower charges, followed by Na, and then by K. Na_4_V_2_(PO_4_)_3_ and Li_4_V_2_(PO_4_)_3_ systems adopted averaged OSs and C2 SGs in the ground state and showed small band gaps of 0.13 eV and 0.20 eV, respectively (Table 1). K_4_V_2_(PO_4_)_3_ in the ground state, identically to Na_4_Ti_2_(PO_4_)_3_ [13], adopted a mixed OS but featured an C2 SG with a band gap of 0.76 eV. The most interesting feature of this study is that any studied symmetry increase in Li_4_V_2_(PO_4_)_3_ either to the SG R32 or $R\bar{3}c$ showed a closing of the band gap. However, Na_4_V_2_(PO_4_)_3_ demonstrated the presence of a non-zero band gap for the averaged OS in the R32 SG and a zero band gap for averaged OS in the $R\bar{3}c$ SG. In contrast to Na_4_V_2_(PO_4_)_3_ and Li_4_V_2_(PO_4_)_3_, K_4_V_2_(PO_4_)_3_ had a finite band gap in all studied symmetry configurations and vanadium OSs (Table 1). Hereafter, we refer to systems with zero band gaps as metallic.

**Figure 2:**
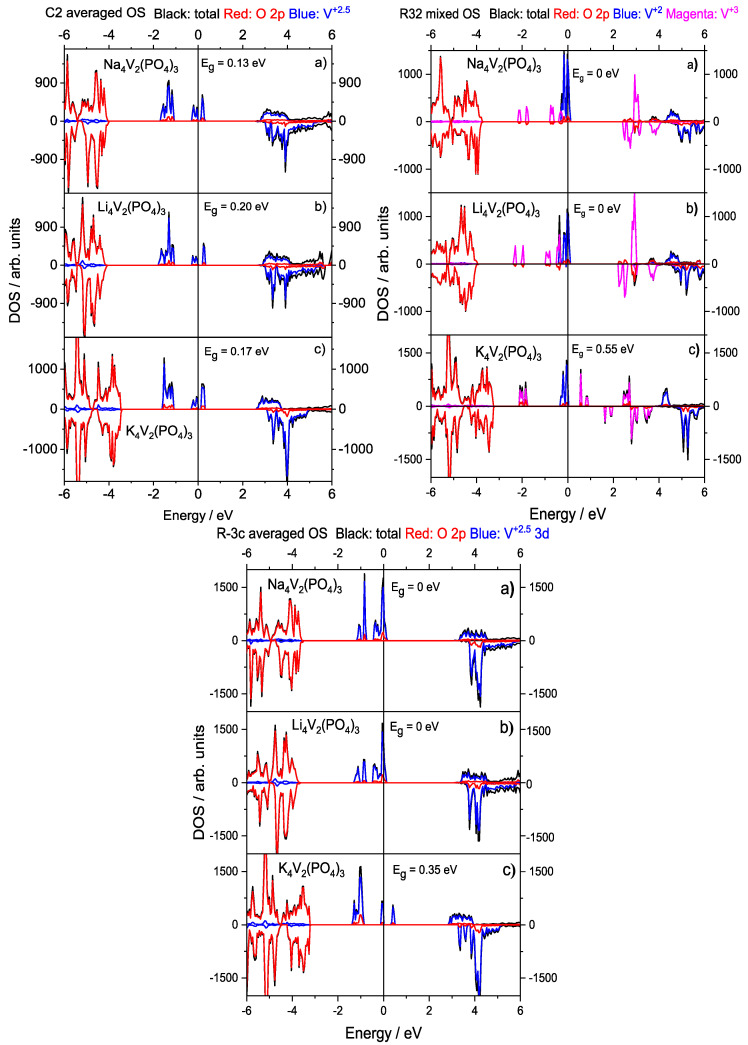
Total and atom projected DOS for (**top left**) C2 averaged V${}^{+2.5}$ OS, (**top right**) R32 mixed V${}^{+2}$/V${}^{+3}$ OS, and (**bottom**) $R\bar{3}c$ averaged V${}^{+2.5}$ OS in (a) Na_4_V_2_(PO_4_)_3_, (b) Li_4_V_2_(PO_4_)_3_, and (c) K_4_V_2_(PO_4_)_3_. Positive and negative DOS values denote the spin-up and spin-down states, respectively. Fermi energy is taken as 0.

The calculated DOSs for the A_4_V_2_(PO_4_)_3_ systems are presented in Figure 2. In the following, we only compared the following cases, namely, those with high symmetry $R\bar{3}c$ SGs and averaged V${}^{+2.5}$ OSs, mixed V${}^{+2}$/V${}^{+3}$ OSs in an R32 SG (Figure 2), and averaged OSs in a monoclinic C2 SG. One can see that bands close to the Fermi level in all A_4_V_2_(PO_4_)_3_ systems were formed by V 3d-states. Crystal Orbital Overlap Population (COOP) analysis was also performed in order to analyze the bonding character of these states. Identically to our previous results on Na_2_VTi(PO_4_)_3_, COOP analysis showed the states close to the Fermi level to have mostly anti-bonding character in all studied A_4_V_2_(PO_4_)_3_ systems as well [30]. The band gap, if present, was of a d-d character and appeared due to a separate band formed by empty V 3d-states in the spin-up channel (Figure 2, top-left).

This band shifted to lower energies and crossed the Fermi level in Li_4_V_2_(PO_4_)_3_ and Na_4_V_2_(PO_4_)_3_ for the mixed vanadium OSs in the high symmetry R32 structures (Figure 2, top-right). In contrast, the separate empty band remained in K_4_V_2_(PO_4_)_3_ which showed a finite band gap remaining in all configurations such as the averaged OS C2 (0.17 eV), mixed OS R32 (0.55 eV), and averaged OS $R\bar{3}c$ (0.35 eV). Notice that the DOS for the ground state of K_4_V_2_(PO_4_)_3_ with the mixed OS in the C2 SG is not shown. We found its DOS to be identical to the one of the R32 SG.

In Figure 2, one can see that, in the case of a mixed OS K_4_V_2_(PO_4_)_3_ system, the empty conduction band was formed by V${}^{+3}$ states right above the Fermi energy at 0.55 eV. In contrast, neither Li_4_V_2_(PO_4_)_3_) nor Na_4_V_2_(PO_4_)_3_) demonstrated the presence of a separate empty band above the Fermi energy and remained metallic in the mixed OS configurations. However, the reduction of symmetry down to the monoclinic C2 structure led to the appearance of an empty band, as well as the formation of a small gap in the latter two systems. Although the closing of band gap and the appearance of a metallic state are commonly attributed to a stronger delocalization of 3d electrons, our results showed it was only partially valid in these compounds. Our results also showed that Li- and Na-based systems showed almost identical electronic structures in Figure 2. Neither the ionic size nor the electronic configuration such as the presence of p-electrons seemed to have any significant effect on the band structure or the appearance of metallicity in Li_4_V_2_(PO_4_)_3_ and Na_4_V_2_(PO_4_)_3_. Only the introduction of larger K ions destroyed the metallic state in A_4_V_2_(PO_4_)_3_ and resulted in marked structural changes.

The calculated DOSs could at least partially explain why the average OS was preferred over the mixed OS in Na_4_V_2_(PO_4_)_3_ and Li_4_V_2_(PO_4_)_3_. It is due to occupied V${}^{+3}$ states intermixing with occupied V${}^{+2}$ states in the energy range between −1 eV and the Fermi energy, which is energetically preferred (Figure 2 (top right)). Even though Na_4_V_2_(PO_4_)_3_ demonstrates the non-zero band gap for the averaged OS in the R32 SG, the intermixing of states is still an important property. On the contrary, K_4_V_2_(PO_4_)_3_ did not demonstrate the same intermixing because occupied V${}^{+2}$ and V${}^{+3}$ bands were well separated in this structure.

**Figure 3:**
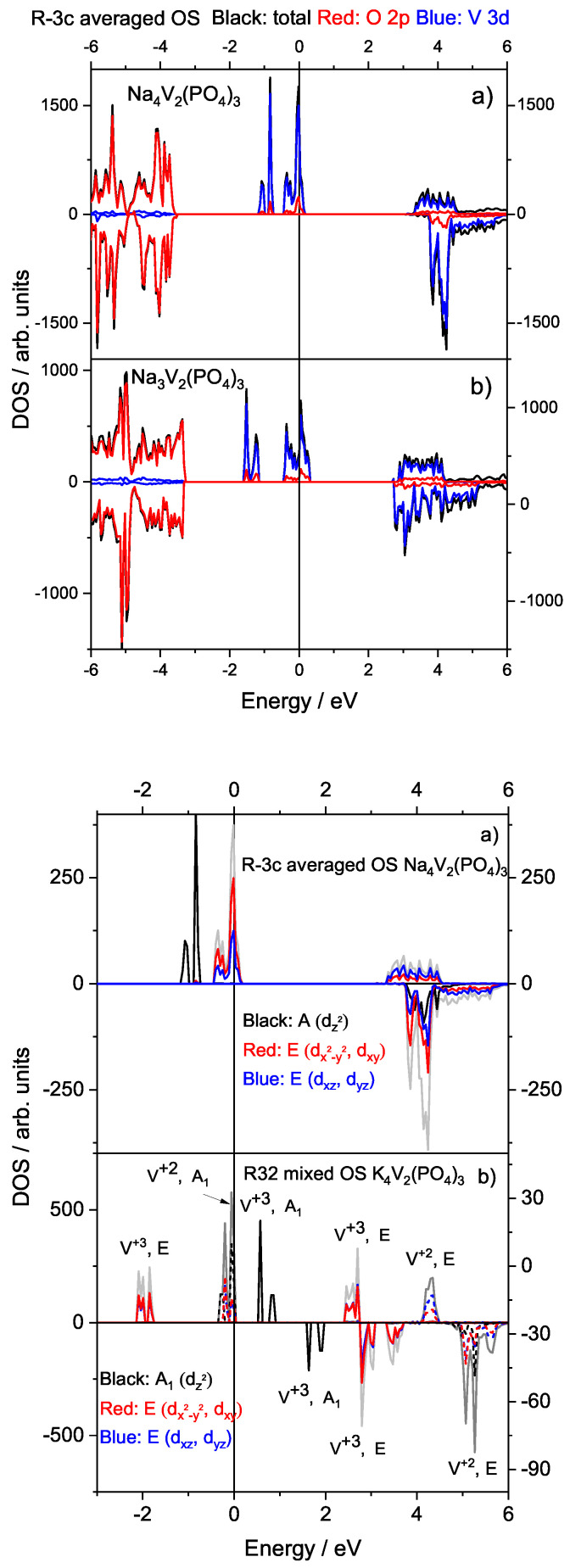
(**top**) Total and atom projected DOS for (a) Na_4_V_2_(PO_4_)_3_ and (b) Na_3_V_2_(PO_4_)_3_ with the averaged OS in the $R\bar{3}c$ SG. (**bottom**) Orbital DOS projections based on the symmetry-allowed irreducible representations for (a) Na_4_V_2_(PO_4_)_3_ with averaged OS and $R\bar{3}c$ SG, and (b) K_4_V_2_(PO_4_)_3_ with mixed OS and R32 SG. The Fermi energy is taken as 0. Dashed lines represent +2 states only.

Another interesting observation of this work was the analysis of a parent Na_3_V_2_(PO_4_)_3_ composition. The typical structure has all M1 (1/4 of total) sodium sites occupied, and these sodium atoms are not (electro)chemically exchangeable. The remaining 2/4 of Na atoms are distributed over 3 M2 sites, with some ordering observed at low temperatures [12,15]. In this work, we constructed a hypothetical rhombohedral Na_3_V_2_(PO_4_)_3_ with an $R\bar{3}c$ SG. For this, we used pure symmetry considerations by removing Na atoms from M1 site (Wyckoff position 6b) in Na_4_V_2_(PO_4_)_3_. In this way, rhombohedral symmetry was maintained, and all M2 positions were fully occupied. The DOS analysis in Figure 3 (top) shows that this particular configuration of Na_3_V_2_(PO_4_)_3_ with high $R\bar{3}c$ symmetry and an averaged V${}^{+2.5}$ OS was also metallic. Moreover, its band electronic structure was almost identical to that of Na_4_V_2_(PO_4_)_3_ in the same symmetry and OS structure. These results indicate that the full occupancy of M2 sites in Na_3_V_2_(PO_4_)_3_ tends to close the band gap.

The structure of the most important bands close to the Fermi energy could be also analyzed in terms of DOS projections on the symmetry-allowed irreducible representations (Figure 3 (bottom)). In accordance with the local V site symmetry with point group $C_{3}$ in both R32 and $R\bar{3}c$ SGs of A_4_V_2_(PO_4_)_3_, the 3d orbitals split into a non-degenerate A(d${}_{z^{2}}$) orbital in $R\bar{3}c$ (A${}_{1}$(d${}_{z^{2}}$) orbital in R32) and two two-fold degenerate E(d${}_{xz}$, d${}_{yz}$) and E(d${}_{x^{2}-y^{2}}$, d${}_{xy}$) orbitals. The A and E orbitals form separate bands which is reflected in the calculated DOS.

For Na_4_V_2_(PO_4_)_3_ in the $R\bar{3}c$ SG, the two two-fold degenerate E orbitals comprise the band at the Fermi energy, and the empty states just above it (metallic state). Obviously, the occupation of E orbitals is possible not only due to symmetry properties but also due to delocalization effects as discussed above. As vanadium is octahedrally coordinated by oxygens in [VO_6_], the second band comprises only the non-degenerate A-orbital, which most likely interacts with the O 2p${}_{z}$ orbital (Figure 3). In the high symmetry $R\bar{3}c$ SG structure, the two-fold degenerate E orbitals interact with O 2p${}_{x}$ and 2$p_{y}$ orbitals. Moreover, the bottom of the conduction band mostly comprises two two-fold degenerate E orbitals.

The structure of the orbital-projected DOS was quite different in the case of K_4_V_2_(PO_4_)_3_ with the mixed OS in the R32 SG. Only two important features are common for the average OS $R\bar{3}c$ of Na_4_V_2_(PO_4_)_3_ and K_4_V_2_(PO_4_)_3_: (i) in accordance with the rhombohedral symmetry, the two degenerate E orbitals are intermixed, and (ii) for the same symmetry reasons, the degenerate and non-degenerate orbitals are clearly separated. Obviously, some additional bands appear due to the separation of V${}^{+2}$ and V${}^{+3}$ 3d electrons in the mixed OS, thereby doubling the number of electronic bands. In the mixed OS K_4_V_2_(PO_4_)_3_ case, it is mostly the non-degenerate A${}_{1}$ orbitals contributing to the top of the valence band. Moreover, this separation between the A_1_ orbitals of V${}^{+2}$ and V${}^{+3}$ states leads to the opening of a 0.55 eV band gap in the R32 SG case (Figure 3). The occupied and unoccupied degenerate V${}^{+3}$ state E orbitals lay at ∼−1.8 eV and at 2.3–3.9 eV from the Fermi level, respectively. In contrast, the degenerate V${}^{+2}$ state E orbitals are unoccupied and lay above 4 eV from the Fermi level.

## 4. Conclusions

1. NASICON-structured Li_4_V_2_(PO_4_)_3_ and Na_4_V_2_(PO_4_)_3_ with fully occupied alkali metal sites adopt monoclinic structures with C2 SGs and averaged vanadium OS V${}^{+2.5}$ in the ground state. On the other hand, K_4_V_2_(PO_4_)_3_ adopted a monoclinic structure with a C2 SG, which was stabilized by the mixed vanadium oxidation state V${}^{+2}$/V${}^{+3}$ in the ground state.
2. Li_4_V_2_(PO_4_)_3_ and Na_4_V_2_(PO_4_)_3_ possesed small band gaps of 0.13 eV and 0.20 eV in the ground state, respectively. However, monoclinic to rhombohedral symmetry increases closed the band gap and demonstrated the possibility of a metallic state in both the V${}^{+2.5}$ averaged (in the $R\bar{3}c$ SG for Na_4_V_2_(PO_4_)_3_ and the $R\bar{3}c$ and R32 SGs for Li_4_V_2_(PO_4_)_3_) and V${}^{+2}$/V${}^{+3}$ mixed vanadium oxidation states. In contrast, K_4_V_2_(PO_4_)_3_ retained a small band gap in all studied structures with different symmetries and vanadium OSs.
3. The band gap in these structures was of a d-d character, independent of the symmetry or vanadium oxidation state. In the case of mixed vanadium oxidation, it was due to an empty band formed by the V${}^{+3}$ states.
4. The special configuration of high $R\bar{3}c$ symmetry Na_3_V_2_(PO_4_)_3_ with Na atoms removed from all M1 sites but completely occupied M2 sites as in initial Na_4_V_2_(PO_4_)_3_ also showed the appearance of a metallic state.
5. The orbital-projected DOS analysis for a rhombohedral symmetry shows that, in an averaged oxidation state for Na_4_V_2_(PO_4_)_3_, the band around the Fermi level is mostly comprised of two-fold degenerate E orbitals, whereas, in the mixed OS, it is mostly the non-degenerate A_1_ orbitals contributing to the top of the valence band. Moreover, the separation between the A_1_ orbitals of V${}^{+2}$ and V${}^{+3}$ states led to the opening of a band gap.
6. These results might contribute to a fundamental understanding of the crystal and electronic structure, as well as provide valuable guidance for researchers performing experimental investigations on this important class of materials.

## Figures and Tables

**Table 1:** The structural properties of A_4_V_2_(PO_4_)_3_ as calculated with the B1WC hybrid functional. *a*,*c* lattice parameters in the hexagonal setting, *q* effective atomic charge of vanadium from Mulliken analysis, μ magnetic moment of vanadium atoms, band gap value $E_{g}$, and total energy difference with respect to the ground state structure are marked in bold. The results for Na_4_V_2_(PO_4_)_3_ and Li_4_V_2_(PO_4_)_3_ with the mixed OS in the C2 SG are not shown, as the energy difference with the R32 SG did not exceed 0.004 eV/cell and was insignificant. Analysis of free parameters in atomic positions is also excluded.

	*a*/Å	*c*/Å	*q*/e	μ/$\mu_{B}$	$E_{g}$/eV	$\Delta E_{\mathrm{tot}}$/eV /Cell
$R\bar{3}c$/Average OS: V${}^{+2.5}$						
Li_4_V_2_(PO_4_)_3_	8.96	19.58	+1.17	2.56	0	0.597
Na_4_V_2_(PO_4_)_3_	8.97	21.15	+1.19	2.57	0	0.595
K_4_V_2_(PO_4_)_3_	9.20	22.33	+1.21	2.55	0.35	0.224
R32/Average OS: V${}^{+2.5}$						
Li_4_V_2_(PO_4_)_3_	8.97	19.49	V${}^{+2.5}$ +1.18	V${}^{+2.5}$ 2.53	0	0.352
			V${}^{+2.5}$ +1.17	V${}^{+2.5}$ 2.56		
Na_4_V_2_(PO_4_)_3_	8.97	21.17	V${}^{+2.5}$ +1.20	V${}^{+2.5}$ 2.53	0.41	0.141
			V${}^{+2.5}$ +1.20	V${}^{+2.5}$ 2.53		
K_4_V_2_(PO_4_)_3_	9.20	22.33	V${}^{+2.5}$ +1.21	V${}^{+2.5}$ 2.55	0.35	0.224
			V${}^{+2.5}$ +1.21	V${}^{+2.5}$ 2.55		
R32/Mixed OS: V${}^{+2}$/V${}^{+3}$						
Li_4_V_2_(PO_4_)_3_	8.97	19.58	V${}^{+2}$ +1.10	V${}^{+2}$ 2.99	0	0.940
			V${}^{+3}$ +1.20	V${}^{+3}$ 2.11		
Na_4_V_2_(PO_4_)_3_	8.98	21.15	V${}^{+2}$ +1.14	V${}^{+2}$ 3.00	0	0.825
			V${}^{+3}$ +1.18	V${}^{+3}$ 2.11		
K_4_V_2_(PO_4_)_3_	9.22	22.38	V${}^{+2}$ +1.15	V${}^{+2}$ 3.00	0.55	0.153
			V${}^{+3}$ +1.23	V${}^{+3}$ 2.04		
C2/Average OS: V${}^{+2.5}$						
**Li_4_V_2_(PO_4_)_3_**	**8.96**	**19.60**	**+1.18**	**2.55**	**0.20**	**0**
	*b* = **9.96**					
**Na_4_V_2_(PO_4_)_3_**	**8.96**	**21.18**	**+1.19**	**2.55**	**0.13**	**0**
	*b* = **9.97**					
K_4_V_2_(PO_4_)_3_	9.24	22.31	+1.20	2.56	0.17	0.200
	*b* = 9.20					
C2/Mixed OS: V${}^{+2}$/V${}^{+3}$						
**K_4_V_2_(PO_4_)_3_**	**9.21**	**22.34**	**V${}^{+2}$ +1.15**	**V${}^{+2}$ 3.00**	**0.76**	**0**
			**V${}^{+3}$ +1.22**	**V${}^{+3}$ 2.07**		

## Data Availability

The data presented in this study are available in the article.

## Abbreviations

The following abbreviations are used in this manuscript:

DFT	Density Functional Theory
LCAO	Linear Combination of Atomic Orbitals
OS	Oxidation State
SG	Space Group
WC	Wu–Cohen
NVP	Na_3_V_2_(PO_4_)_3_
